# Detection of genomic signatures of recent selection in commercial broiler chickens

**DOI:** 10.1186/s12863-016-0430-1

**Published:** 2016-08-26

**Authors:** Weixuan Fu, William R Lee, Behnam Abasht

**Affiliations:** 1 Department of Animal and Food Sciences, University of Delaware, Newark, DE 19716 USA; 2 Maple Leaf Farms, Inc, Leesburg, IN 46538 USA

**Keywords:** Chickens, Selection signatures, Breeding, Commercial broilers

## Abstract

**Background:**

Identification of the genomic signatures of recent selection may help uncover causal polymorphisms controlling traits relevant to recent decades of selective breeding in livestock. In this study, we aimed at detecting signatures of recent selection in commercial broiler chickens using genotype information from single nucleotide polymorphisms (SNPs). A total of 565 chickens from five commercial purebred lines, including three broiler sire (male) lines and two broiler dam (female) lines, were genotyped using the 60K SNP Illumina iSelect chicken array. To detect genomic signatures of recent selection, we applied two methods based on population comparison, cross-population extended haplotype homozygosity (XP-EHH) and cross-population composite likelihood ratio (XP-CLR), and further analyzed the results to find genomic regions under recent selection in multiple purebred lines.

**Results:**

A total of 321 candidate selection regions spanning approximately 1.45 % of the chicken genome in each line were detected by consensus of results of both XP-EHH and XP-CLR methods. To minimize false discovery due to genetic drift, only 42 of the candidate selection regions that were shared by 2 or more purebred lines were considered as high-confidence selection regions in the study. Of these 42 regions, 20 were 50 kb or less while 4 regions were larger than 0.5 Mb. In total, 91 genes could be found in the 42 regions, among which 19 regions contained only 1 or 2 genes, and 9 regions were located at gene deserts.

**Conclusions:**

Our results provide a genome-wide scan of recent selection signatures in five purebred lines of commercial broiler chickens. We found several candidate genes for recent selection in multiple lines, such as *SOX6* (*Sex Determining Region Y-Box 6*) and *cTR* (*Thyroid hormone receptor beta*). These genes may have been under recent selection due to their essential roles in growth, development and reproduction in chickens*.* Furthermore, our results suggest that in some candidate regions, the same or opposite alleles have been under recent selection in multiple lines. Most of the candidate genes in the selection regions are novel, and as such they should be of great interest for future research into the genetic architecture of traits relevant to modern broiler breeding.

**Electronic supplementary material:**

The online version of this article (doi:10.1186/s12863-016-0430-1) contains supplementary material, which is available to authorized users.

## Background

Artificial selection is the primary factor in the domestication and breeding history of livestock species. Modern broiler (meat-type) chickens have been under strong artificial selection, mostly for traits of economic importance for farmers, such as growth rate, feed efficiency and body composition [1]. Comparing a modern broiler chicken cross, Ross 308, with a broiler population that had not been subjected to artificial selection since 1957 [i.e., Athens-Canadian Random-bred Control (ACRBC) strain], Havenstein *et al.* (2003) found that the average body weight at 42 days of age increased from 539 g in 1957 as represented by the ACRBC strain to 2,672 g in 2001 as represented by the Ross 308 strain and that the feed conversion ratio decreased from 2.34 to 1.43 over the same time period [2]. The authors indicated that genetic selection contributed 85–90 % of the improvement in growth rate over the past 45 years. These dramatic phenotypic changes imply that the frequencies of the underlying causal polymorphisms themselves have been altered by selection for performance in these traits during the intervening time period. Thus, detecting the genomic footprints of artificial selection should help researchers to identify the causal polymorphisms underlying phenotypic changes and to better understand the biological and genetic mechanism controlling these traits.

In broiler chicken genetic stocks, the traits of most relevance for recent decades of breeding, such as feed efficiency, growth rate and meat yield, are complex traits that are controlled by many genes. Consequently, it is highly likely that selection for these traits has worked simultaneously on multiple causal genes across the genome. Therefore, high throughput methods are required to screen the whole genome for signatures of recent selection. With the availability of high throughput genotyping tools, such as high-density SNP arrays and next-generation sequencing, it has become possible to conduct genome-wide studies for detection of genomic footprints of artificial selection.

Using whole-genome re-sequencing and high-density SNP chips, respectively, Rubin *et al.* (2010) and Elferink *et al.* (2012) have investigated selection signatures in large numbers of chicken breeds using Z-transformed pooled heterozygosity (ZHp) scores. This statistic estimates local heterozygosity depression in chromosomal regions [3, 4] and has been appropriately applied for detecting alleles that have swept to fixation or near-fixation by long-term directional selection or during domestication [5]. However, modern broiler chicken breeding practices that have a more recent selection history, and have been employed to select for a suite of more sophisticated traits, such as feed efficiency and meat yield with different selection priorities in different specialized component lines, would not be expected to leave such common and distinct changes. Therefore, most signatures of more recent selection are likely yet to be uncovered in the genome of modern broiler chickens.

In contrast with the ZHp method, it has been suggested that methods based on extended haplotype homozygosity (EHH) [6] or change in allele frequency spectrum can be more useful for detecting signatures of recent selection in animal breeds [5, 7]. These population genetics methods are developed to find frequent alleles and long-range haplotypes with high frequency, which are indicatives of chromosomal regions under recent selection [6]. In dairy cattle, Qanbari *et al.* (2010) adopted the relative EHH method to detect signatures of positive selection in Holstein–Friesian cattle using a 50K SNP array [7]. Zhang *et al.* (2012) applied the same method to detect selection signatures in two broiler chicken lines divergently selected for abdominal fat content and reported the *PC1/PCSK1* region as the most likely candidate region to have a causal effect on abdominal fat weight [8, 9]. To detect selection signatures in Fleckvieh cattle, Qanbari *et al.* (2014) applied two statistical methods, the integrated haplotype score (iHS) [10] and the composite likelihood ratio (CLR) [11, 12], and found that many candidate regions were relevant to coat coloring pattern, neurobehavioral functioning and sensory perception [13].

Concerned that the EHH and iHS methods may have insufficient power to identify the alleles that have been more recently strongly selected and swept to near-fixation or fixation [14], Sabeti *et al.* (2007) developed the cross-population extended haplotype homozygosity (XP-EHH) test to detect signatures of recent selection by comparing EHH between two different populations regardless of whether or not the favored allele had reached fixation. Also, the single-population CLR method does not take advantage of larger differences in allele frequencies between two breeds and is very sensitive to SNP ascertainment bias. To overcome these limitations, Chen *et al.* (2010) developed the cross-population composite likelihood ratio (XP-CLR) test [15].

In this study, we applied both XP-EHH and XP-CLR methods to five commercial broiler purebred populations, including three male lines and two female lines, to detect the signatures of recent selection in these commercial broiler stocks. The findings here help to improve our understanding of the biological mechanisms controlling economically important traits in modern commercial broiler chickens.

## Methods

### Animals and data preparation

A total of 565 chickens from five commercial purebred lines were genotyped using the 60K SNP Illumina iSelect chicken array [16]. Blood samples were collected from a wing vein and the samples were shipped on dry ice to DNA LandMarks (Saint-Jean-sur-Richelieu, Quebec, Canada) for DNA extraction and genotyping with the 60K SNP Illumina iSelect chicken array. All genotyped birds were males and were sampled from 3 male (broiler sire) lines, ML1, ML2 and ML3, and 2 female (broiler dam) lines, FL1 and FL2. In total, 318 birds were sampled from male lines: 24 ML1, 256 ML2 and 38 ML3 chickens; and 247 birds were sampled from female lines: 126 FL1 and 121 FL2 chickens. The FL1, FL2 and ML1 chickens as well as a portion of ML2 (ML2_0; n = 96) chickens were elite sires randomly sampled from three overlapping generations. Another portion of ML2 genotyped chickens (ML2_1; n = 160) was a random sample of the progeny of the ML2_0 elite sires. The ML3 genotyped chickens were random samples of male chickens from this purebred population. Male and female lines originated from different breeds, i.e. the male line from Cornish, a meat type breed, and the female lines from White Rock, a dual-purpose breed. Each of these five lines came from a different source to Heritage Breeders, and all lines, except FL1, have been reproductively isolated for more than 40 generations. A one-time crossbreeding with ML2 and then backcrossing with FL1 happened early in the history of FL1, and the resulting new FL1 population has been reproductively isolated for more than 25 generations. In each generation within each purebred line, approximately 50 to 80 male and 500 to 800 female birds have been selected for reproducing the next generation. More details about genetic diversity in ML2, FL1 and FL2 can be found in a previous study, where the three lines were labeled as B, C and D, respectively [17]. Since allele frequency of SNPs and linkage disequilibrium (LD) among SNPs were highly consistent between the two sampled generations of ML2, ML2_0 and ML2_1 (unpublished data in our laboratory) and the methods we used for detecting recent selection signatures relied on allele frequency and LD information, we combined data from ML2_0 and ML2_1 in the current study.

Although male lines (ML1, ML2 and ML3) have shared similar selection objectives, which have been primarily focused on increasing growth rate, feed efficiency and breast muscle yield, the relative magnitude of selection pressure on these major traits varied among the three lines: ML1 has been more heavily selected for rapid growth, ML2 for high breast meat yield and ML3 for improved feed efficiency. Therefore, we expect artificial selection has unequally increased the frequency of alleles controlling these traits among the male lines, and some alleles may have been selected in opposite direction between male and female lines. The expected differences in the allele frequency among these purebred lines make it possible to apply the two population comparison based methods, XP-EHH and XP-CLR, in our study.

The 60K SNP Illumina iSelect chicken array contains a total of 57,636 SNPs [16]. For the purpose of this study, we used only SNPs with assigned positions on the current chicken genome based on the latest reference genome (Gallus gallus 4.0 UCSC, May 2012). We excluded SNPs with a call rate < 90 % or Mendelian inconsistency > 0.001 and SNPs that were monomorphic among all the purebred lines. We also excluded SNPs on chromosomes 16 and W and two linkage groups, as there were too few SNPs in the 60K SNP Illumina iSelect chicken array for these chromosomes. After quality control, 48,950 SNPs were used in subsequent analyses of the five populations (Table 1).

**Table 1:** Quality control of genotype data

Total SNPs	Quality control					SNPs used
	NI^1^	MI^2^	UG^3^	MO^4^	LCR^5^	
57,636	1,507	1,478	873	4,292	536	48,950

Note: ^1^SNPs on GGA16, W and two linkage groups (LGE22C19W28_E50C23 and LEG64) or SNPs with unknown positions on Galgal4; ^2^SNPs with Mendelian inconsistency; ^3^Ungenotyped SNPs; ^4^SNPs monomorphic among all the 5 purebred lines; ^5^SNPs with low call rate

Since a linkage map was required for the XP-CLR method, we calculated the genetic positions of all the markers in the 60K SNP Illumina iSelect chicken array using a subset of markers with known genetic positions based on the male linkage map previously provided by Groenen *et al.* [18]), and assuming that the recombination rates between two markers were uniformly distributed. We used BEAGLE (Version 3.3.2) [19] to impute missing genotypes, phase the chromosomes and identify haplotype structure at the candidate selection regions in each purebred line.

### The XP-EHH test

The XP-EHH test uses the integrated EHH (*iHH*) of a core SNP in two populations, A and B, rather than two alleles in a single population. The unstandardized XP-EHH statistic can be calculated as [14]:

1 $$ \mathrm{unstandardized}\;XP-EHH= \ln\;\left(\frac{iH{H}_A}{iH{H}_B}\right) $$

where *iHH*_*A*_ and *iHH*_*B*_ are the integrated EHH of a given core SNP in population A and B, respectively. A large positive value of XP-EHH suggests either selection in population A or a negative value in population B.

We used the software developed by Pickrell *et al.* [20] to estimate unstandardized XP-EHH statistics for all SNPs (after quality control) in all five purebred lines with cross-population comparison of each purebred line with the four remaining lines: for example, ML1 *vs.* ML2, ML3, FL1 or FL2 (four cross-population tests for each line). The unstandardized XP-EHH statistics were standardized using their means and variances in each purebred comparison. Because previous studies found that the standardized XP-EHH statistics follows the standard normal distribution [14, 21, 22], *P*-values of SNPs were estimated using the standard normal distribution. For each purebred comparison, we determined the candidate regions under positive selection by clustering the significant core SNPs (*P-*value < 0.05) with a distance of less than 200 kb.

### The XP-CLR test

To confirm selection signatures detected by the XP-EHH analysis, we applied the XP-CLR test based on the change in the allele frequency spectrum, since it has the advantage of enlarging signals to allow the resolution of more precise regions [15]. The XP-CLR test [15] was also adopted for the five purebred lines by cross-population comparison of each line with the four remaining lines as reference populations using the XP-CLR 1.0 software available at http://genetics.med.harvard.edu/reich/Reich_Lab/Software.html (last accessed Jan. 3, 2016). The grid points at the putative selected allele positions were set along each chicken chromosome with a spacing of 2 kb, and sliding window size was set as 0.5 cM around the grid points. To reduce the contribution of SNPs in high LD to the likelihood function, the cut-off level of absolute pairwise correlation coefficient of two SNPs was set to 0.9 for estimation of the weight factor (*w* [15]).

For each cross-population comparison, the cutoff threshold of 0.5 % XP-CLR scores was applied to determine windows with strong signals across the whole genome. We then determined the candidate selection regions by clustering these windows, such that windows with genetic distances less than 1 cM constitute a candidate selection region. The selection regions detected by both statistical methods for each purebred line were determined as candidate regions under positive selection. A Karyogram layout of candidate selection regions detected by both tests was created using the ggbio R package [23].

In comparison with human populations, modern livestock breeds generally have much smaller effective population sizes due to animal breeding programs [24–26]. To minimize false discovery due to genetic drift resulted from the small effective population size, the candidate selection regions shared by multiple purebred lines were consider as high-confidence selection regions, and these regions were chosen to identify candidate genes using the genomic database search engine BioMart (http://www.biomart.org/).

## Results

**Fig. 1:**
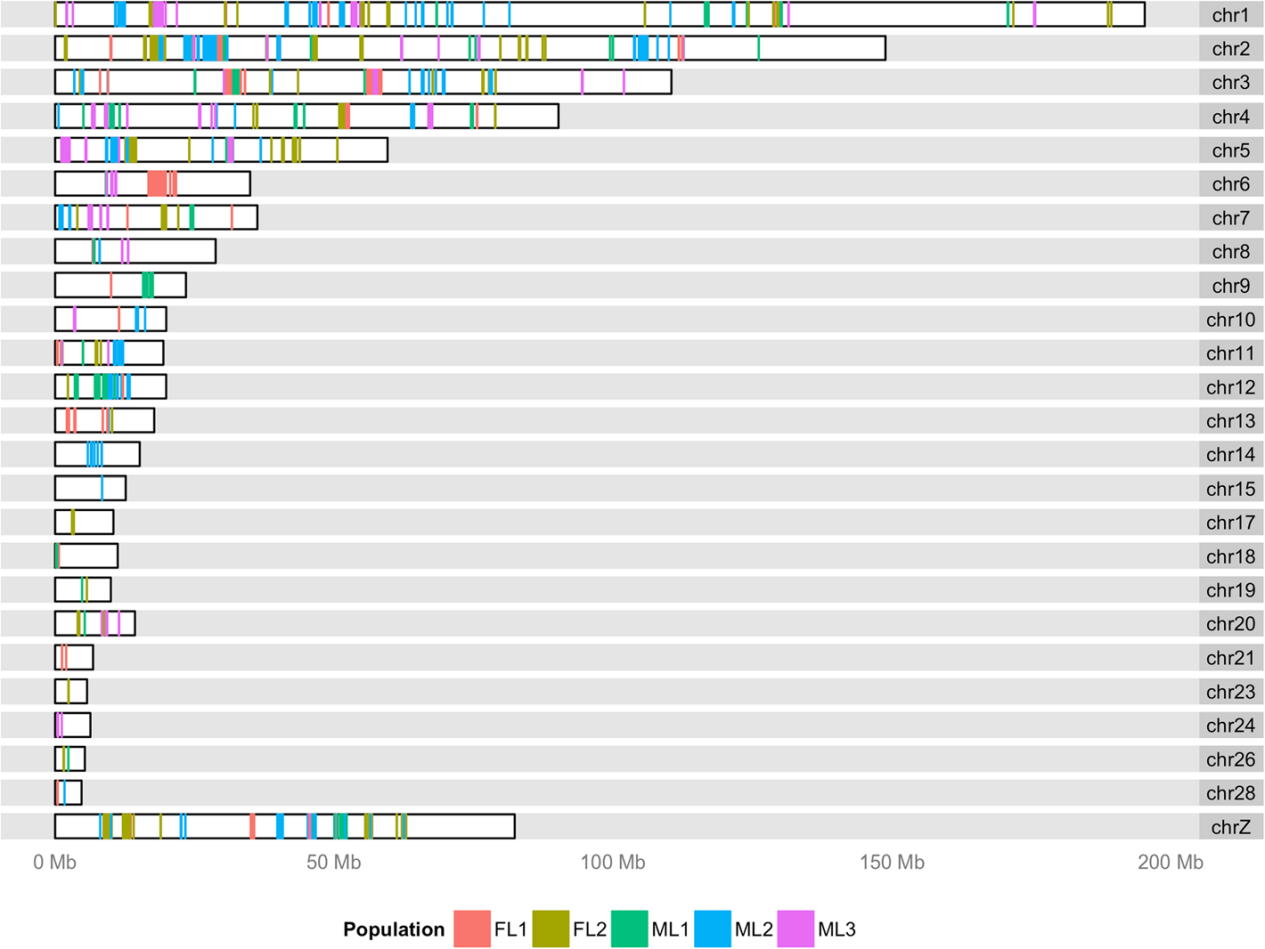
Candidate selection regions detected by XP-EHH and XP-CLR tests. For each purebred line, the overlapped regions detected by the XP-EHH and XP-CLR tests were presented based on the ranges from XP-CLR test. Each population is denoted by a different color

**Fig. 2:**
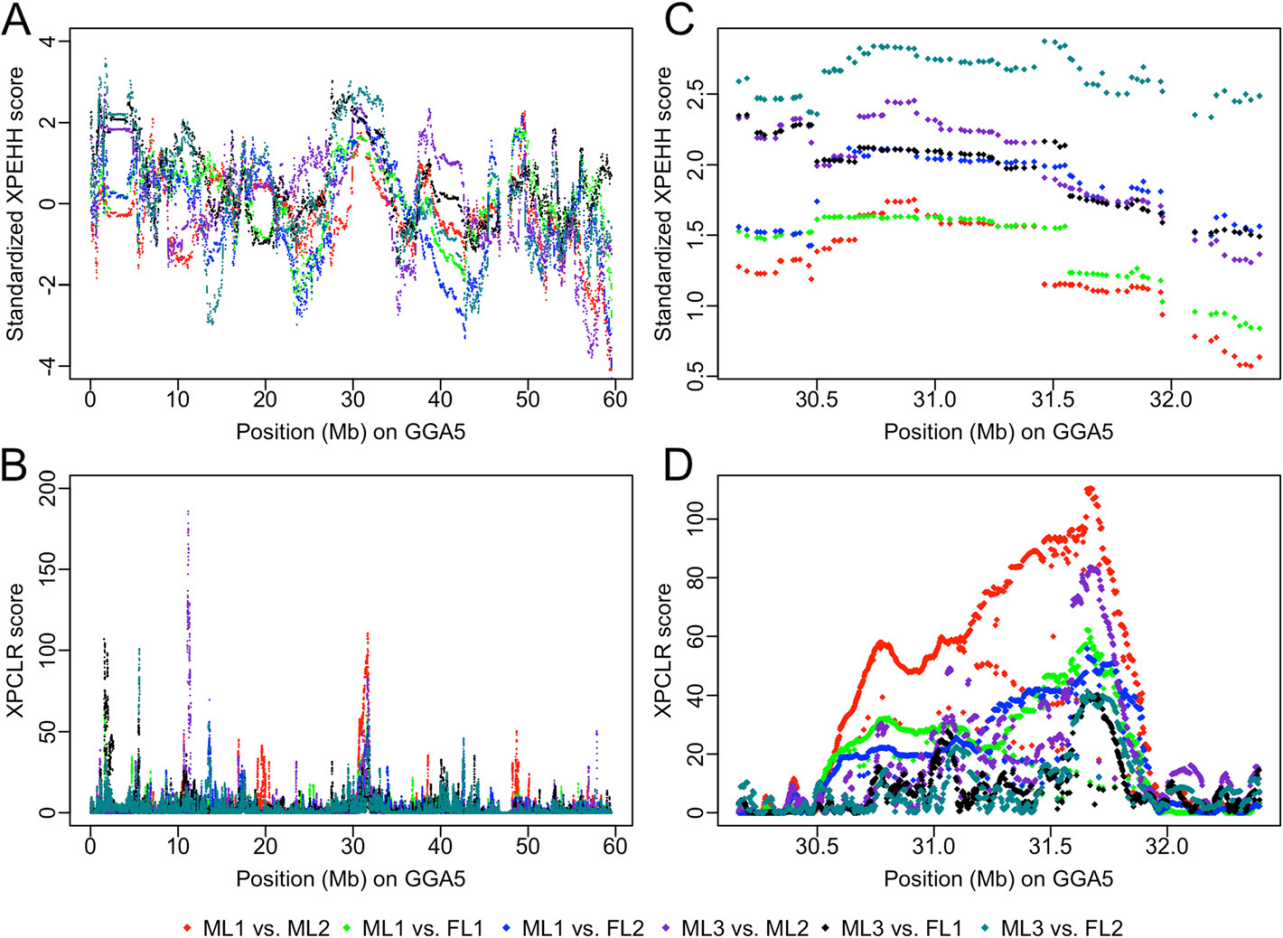
XP-EHH and XP-CLR scores on GGA5. A and B: The results of the XP-EHH (**a**) and XP-CLR (**b**) statistics on the whole GGA5 using multiple population comparisons. **c** and **d**: The results of the XP-EHH (**a**) and XP-CLR (**b**) statistics in a candidate selection region (GGA5: 31.06-31.82 Mb) shared by ML1 and ML3. The dots in the Fig. 2b and 2d represent the XP-CLR scores of sliding windows, and the dots in the Fig. 2a and 2c represent the standardized XP-EHH scores of SNPs. Each comparison of ML1 or ML3 against the other 3 lines is denoted by a different color

In total, 1,079 putative selection regions were detected with *P*-values < 0.05 using XP-EHH test (Additional file 1: Table S1), and 1,018 putative selection regions were detected using the criterion of a 0.5 % cutoff of XP-CLR scores (Additional file 2: Table S2). Regions detected using XP-EHH overlapped 31.53 % of the regions that were identified using XP-CLR (Additional file 3: Table S3). Even though 328 overlapped regions (i.e., detected by both methods) were presented on Additional file 3: Table S3, some regions detected by either the XP-EHH or XP-CLR tests were wide enough to overlap with more than one region detected by the other test. Therefore, in total, 224 and 321 unique regions were detected using XP-EHH and XP-CLR tests, respectively. In each line, approximately 11.09 % of the chicken genome was covered by regions detected by XP-EHH methods, while approximately 2.58 % of the chicken genome was covered by regions detected by XP-CLR methods. The overlapped regions (shared by both methods) only represented approximately 1.45 % of the chicken genome in each line. Selection regions detected by XP-EHH were much wider, mainly because the EHH test is an LD-based method, and LD is expected to extend over longer distances in regions under recent selection [17]. For example, Fig. 2a and 2b represent the results of XP-EHH and XP-CLR tests on GGA5, which show candidate regions detected by XP-CLR tests were overall narrower and perhaps more accurate than those detected by the XP-EHH tests. Thus, to narrow down regions that overlapped between the two methods, we considered the 321 regions based on the XP-CLR test as the candidate selection regions. Their ranges are presented in Additional file 3: Table S3 and visualized in Fig. 1.

By further examining these 321 regions, we identified 42 regions that were shared by two or more purebred lines (Additional file 4: Table S4) and considered them as high-confidence selection regions. Figure 2c and 2d represent the results of XP-EHH and XP-CLR tests in one of the 42 regions (GGA5: 31.06-31.82 Mb) shared by ML1 and ML3. To further narrow down these high-confidence selection regions, only common regions shared by two or more purebred lines were counted in the overlapped regions. Of these 42 common regions, 20 were 50 kb or less while 4 regions were larger than 0.5 Mb. Using BioMart, 91 genes could be found in the 42 regions (Additional file 4: Table S4) among which 9 regions were located at gene deserts and 19 regions only harbored 1 or 2 genes. For the 9 regions located at gene deserts, the genes closest to them (±100 kb) are listed on Additional file 4: Table S4.

**Fig. 3:**
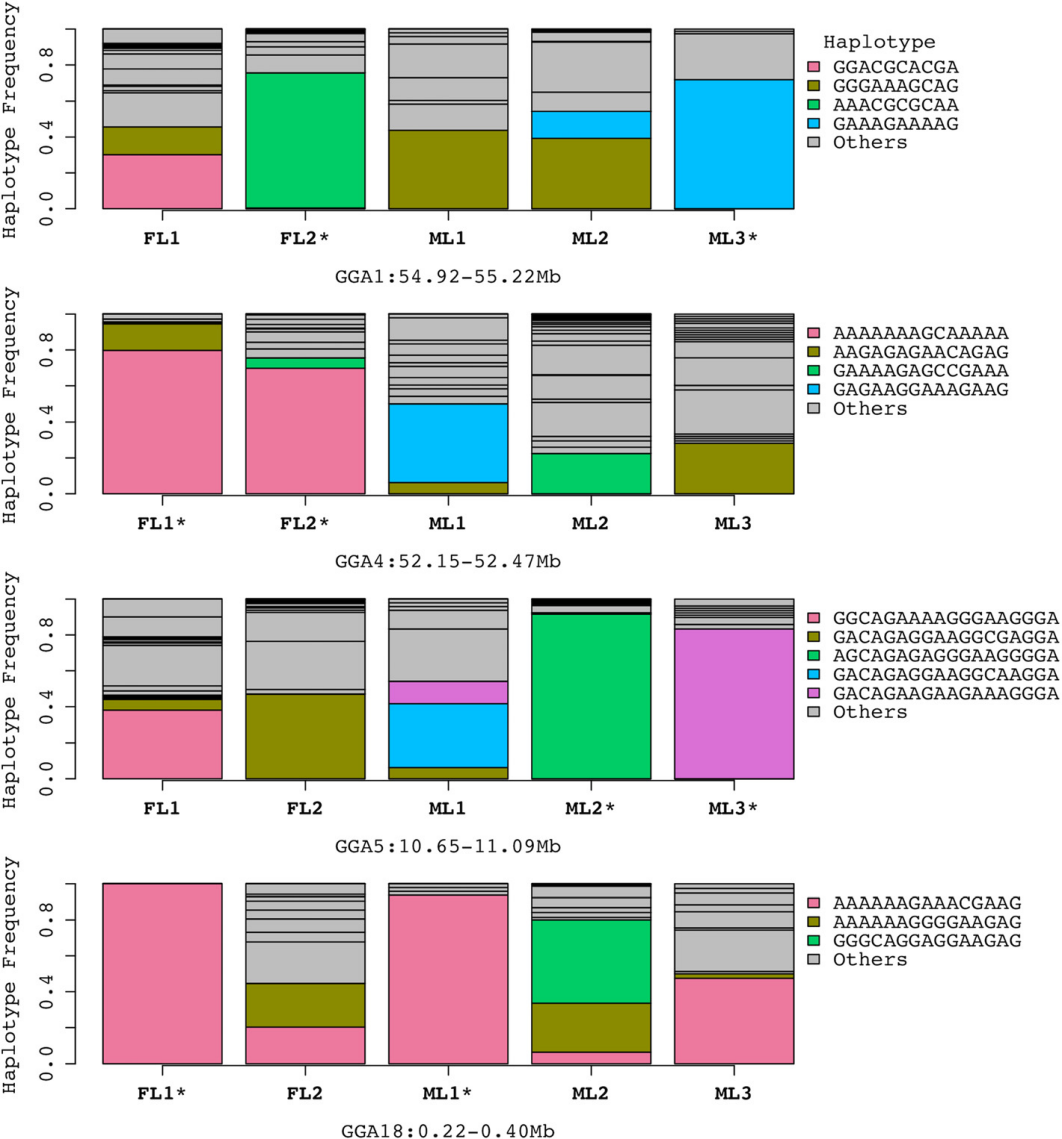
Haplotype frequencies of SNPs in four selection regions detected in multiple purebred lines of broiler chickens. The various colors excluding grey refer to the major haplotypes identified in the 5 purebred lines. At each denoted region, a selection signature was detected in the purebred lines marked with “*”

To gain insight into population differences in the overlapped candidate regions, we constructed haplotypes and estimated haplotype frequencies in these regions in each population (Additional file 5: Table S5). This analysis was performed only for the the high-confidence selection regions that contained at least 5 informative SNPs in our genotype data (14 out of 42 regions). Figure 3 represents the results of haplotype analysis in four selection regions containing 10 to 20 SNPs in our genotype data. As demonstrated in Fig. 3, haplotypes with high frequencies were detected in the denoted genomic regions. For example, in a selection region on GGA4 (52.15-52.47 Mb), the same haplotype showed high frequency in FL1 and FL2, although the range of this region was more than 300 kb. Another interesting example is a ~240 kb region on GGAZ (45.49-45.73 Mb). In this region, all three male lines had the same major haplotype, but each female line had a different major haplotype (Additional file 5: Table S5).

## Discussion

In modern broiler breeding, the practice of selective mating is utilized to influence the expression of economically important traits in subsequent generations. Through such selection, the “beneficial” alleles tend to become more frequent in populations over time. In our study, we applied XP-EHH and XP-CLR tests to detect the genomic regions under recent selection by measuring the characteristics of extended haplotype homozygosity and changes in the allele frequency spectrum. By cross-population comparisons of five commercial broiler purebred lines, we identified the genomic regions that are most likely to harbor genes related to traits of economic importance in broiler chickens.

It should be mentioned that both bottleneck events and genetic drift have potential to influence the results of selection signature studies such as this one. Based on records from Heritage Breeders, bottleneck events did not occur in the lines used in the present study for more than 40 generations. To minimize false discovery due to genetic drift, only 42 of the candidate selection regions that were shared by 2 or more purebred lines were considered as high-confidence selection regions in our study. Another potential limitation is inherent to cross-population methods, which may fail to detect a selection signature where the desirable allele has been under similar level of positive or negative selection pressure in all these purebred lines that were studied. However, this limitation should not be a major concern because the relative magnitude of selection pressure on major traits, growth rate, feed efficiency and breast muscle yield, varied among the 5 purebred lines. Also, unlike in male lines, selection for reproduction traits has been emphasized in female lines.

### Candidate selection regions

We compared the genes in the candidate selection regions in our study with those from two previous studies on detecting selection sweeps in chickens. Among 91 genes in the 42 regions in our study, only two genes (*SOX6* and *GJD2*) are in genomic regions detected by Rubin *et al.* (2010) in commercial broilers. Also, only two genes in our list (*GAS7* and *STXBP6;* Additional file 4: Table S4) are among 366 genes (based on Ensembl gene ID) detected by Elferink *et al.* (2012). This low extent of overlap with previous studies is likely related to the different methods that we used for detecting selection signatures in the present study. As mentioned before, we aimed at detecting signatures of recent selection using the cross-population methods, whereas the ZHp method used in two previous studies is primarily focused on detecting older selection signatures such as those accumulated during domestication. For better comparison, we estimated ZH scores over sliding 5-marker windows on autosomes using data from our study (Additional file 6: Supplemental file). This analysis led to the detection of 41 selection regions containing 81 genes, including 31 genes detected in broilers from two previous studies (Additional file 7: Table S7 and Additional file 8: Table S8) although our resource populations were much different from those in the two previous studies. The most possible reason of high overlap using ZH scores is that some older selection signatures during chicken domestication were shared with commercial broilers used in our study as well as two previous studies.

In our results from two cross-population methods (XP-EHH and XP-CLR), 42 regions were detected by both methods in multiple populations, which might indicate that gene(s) in these regions have been independently selected in multiple populations, i.e., parallel selection. Of these 42 regions, 13 regions were shared among male lines, 5 regions were shared among female lines and 24 regions were shared among both male and female lines. In addition to the significant overlap between the suites of selected traits among all lines selected for broiler performance (especially for growth rate, meat yield, and feed efficiency), the shared selection regions among male and female lines suggests that alleles with pleiotropic effects have been under recent selection in these regions, i.e., alleles that are positively correlated with growth related traits and negatively with reproduction traits, or vice versa. Alternatively, the regions shared by male and female lines may contain closely linked genes impacting both growth and reproduction traits. To further analyze the high-confidence selection regions, we examined population differences in haplotype structure and frequencies and identified the major haplotype (the haplotype with the highest frequency) within each population at each denoted region. The results showed that, in some candidate selection regions, the same major haplotype is shared by multiple lines (Fig. 3 and Additional file 5: Table S5). However, in 9 out of 14 candidate selection regions presented in Additional file 5: Table S5, such as GGA1: 54.92-55.22 Mb and GGA13: 9.38-9.44 Mb, the major haplotype varied greatly among the five purebred lines. The difference in major haplotype may represent high diversity of genetic background among these purebred lines [17]. Alternatively, it is possible that selection has acted on different alleles of a gene in these purebred lines. For example, previous studies found that fertility was reduced in chickens under strong selection for body weight due to the negative genetic correlation between reproduction and growth traits [27–29]. Overall, selection for reproduction traits has been more emphasized in female lines, whereas selection for high feed-efficiency and increased skeletal muscle growth has been the major focus in male lines. Thus, the frequency of alleles benefiting reproduction traits but adversely affecting growth traits are expected to be relatively higher in female lines as compared with the male lines. Some candidate genes with potential pleiotropic effects, such as *STXBP6* and *cTR,* in the high-confidence selection regions are discussed below.

### Candidate genes in regions detected in mulitple populations

In the 42 high-confidence candidate selection regions detected by both methods (XP-EHH and XP-CLR) in two or more purebred lines, we identified several genes related to growth, development, feed efficiency and reproduction in chickens (Table 2). Only a few of them are mentioned below to discuss their potential involvements in controlling these traits.

**Table 2:** A partial list of candidate genes in or near the 42 high-confidence selection regions detected in multiple purebred lines of broiler chickens

Gene name	Gene symbol	Function or association
*Thyroid hormone receptor beta*	*cTR*	Growth, development and homeostasis
*Sex Determining Region Y-Box 6*	*SOX6*	Development of chondrocytes and skeletal muscle
*Actin, alpha, cardiac muscle 1*	*ACTC1*	Muscle development
*Syntaxin binding protein 6*	*STXBP6*	Bone allocation and fecundity traits
*Myosin heavy chain 13*	*MYH13*	Skeletal muscle development
*Calpastatin*	CAST*	Growth and meat quality

Note: *this gene is located close to a candidate selection region detected in a gene desert area on *GGAZ*. Information about chromosomal locations of the 42 candidate selection regions and the full list of candidate genes in or near these regions can be found in Additional file 4: Table S4

#### Myosin heavy chain 13 (MYH13)

*MYH13* is located in a candidate selection region on GGA18 (0.22-0.40 Mb), and other four genes of the *myosin heavy chain* (MyHC) family (*MYH1A, MYH1B, MYH1C, MYH1E*) are located very close to this region. Previous studies found that MyHC genes play important roles in skeletal muscle development [30–32], and the polymorphisms in *MYH3* were significantly associated with growth and body composition traits in Qinchuan cattle [33, 34].

#### Sex determining region Y-Box 6 (SOX6)

*SOX6* is located in a candidate selection region (GGA5: 10.65-11.09 Mb) detected in two male lines, ML2 and ML3. This gene encodes a Sry-related transcription factor that promotes early chondroblast differentiation and plays a critical role in differentiation and proliferation of chondrocytes as well as normal fiber type differentiation of fetal skeletal muscle in mice [35–37].

#### Proprotein convertase subtilisin/kexin type 1 (PCSK1) and calpastatin (CAST)

Although no gene was found inside a candidate selection region around 56.76 Mb on GGAZ due to its small size (8 kb), this region is consistent with findings from two previous studies [8, 9] in which a selection signature was detected using two chicken lines divergently selected for abdominal fat content for 11 generations. Of note, a previously known candidate gene for fatness in chickens, *PCSK1* [8]*,* is located close to this region. Another gene close to this region is *CAST*, which encodes calpastatin, a specific inhibitor of an endogenous calpain. The calpain family plays an important role in embryonic development and muscle growth [38–40]. Many studies have found that polymorphisms in *CAST* are significantly associated with growth traits and meat quality traits in livestock animals [41–46].

#### Actin, alpha, cardiac muscle 1(ACTC1) and Syntaxin binding protein 6 (STXBP6)

Another muscle-related gene, *ACTC1*, was found in one of the selection regions on GGA5 (31.06-31.82 Mb, Fig. 3). This gene encodes cardiac muscle alpha actin in chickens and plays an important role in fetal development as well as cell survival, differentiation and development of muscle [47–50]. *STXBP6* is another gene in this candidate selection region on GGA5. A previous study has indicated *STXBP6* had potential pleiotropic effect on bone tissue and fecundity traits in chickens [51]. Interestingly, this selection sweep, which was detected in two male lines (ML1 and ML3), was also found in a previous study in table egg layer breeds of chickens [52]. One possible reason why this selection sweep is shared by broiler (meat-type) and layer (egg-type) chickens is that both genes, *ACTC1* and *STXBP6*, may influence body weight in broilers and in layers. It should be mentioned that breeders improved meat production in broilers by selection on high body weight at an early age (<8 weeks of age) while they improved feed efficiency and egg production in layers by selection on low body weight at a late age (>24 weeks of age) [53–57]. Alternatively, this shared selection sweep may be explained by the pleiotropic effect of *STXBP6* on both bone tissue and fecundity traits.

#### Thyroid hormone receptor beta (cTR)

*cTR* was found in a selection region on GGA2 (37.90-38.07 Mb)*.* Thyroid hormone can regulate animal growth, development and homeostasis [58], and its receptor mediates thyroid hormone actions [59]. Mice with homozygous mutant *cTR* gene manifest low weight gain and decreased bone development compared to normal mice [60]. In a ~40 kb-length candidate selection region (GGA2: 38.03-38.07 Mb) which was detected in 4 purebred lines (FL1, FL2, ML2 and ML3), there are 3 SNPs in our dataset, which construct the same major haplotype (AAA) in two female lines and ML2, but the major haplotype (GGG) in ML3 is completely different. It should be mentioned that among the five purebred lines of chickens used in our study, ML3 is the most feed-efficient line. It is possible that in this candidate region, the same allele of *cTR* has been selected in two female lines and ML2 while an alternative allele has been selected in ML3. This assumption may be further supported considering diverse functions of thyroid hormone: it has been reported that thyroid hormone also plays a critical role in fertility, but excessive amounts of this hormone in hyperthyroidism has a negative effect on reproduction in humans [61, 62]. Therefore, the pleiotropic effects of thyroid hormone on reproduction and growth traits may explain why the receptor gene, *cTR*, may have been under selection among both female and male lines.

## Conclusions

In this study, we identified novel candidate regions for recent selection in broiler chickens. Based on the biological function of genes in the candidate regions, several genes, such as *SOX6* and *cTR,* have possibly made large contributions to economically important traits in chickens. Our findings suggest that recent selection in broiler breeding has had large impact on frequency of genes controlling economically important traits, such as weight gain, muscle mass, feed efficiency and reproduction. Finally, since most of the candidate genes identified in the present study are novel and have probably been under recent selection, they should be of great interest for future research into the genetic architecture of traits relevant to modern broiler breeding.

## Additional files

Additional file 1:
**Table S1.** Putative selection regions detected by XP-EHH test (XLSX 99 kb) Additional file 2:
**Table S2.** Putative selection regions detected by XP-CLR test (XLSX 92 kb) Additional file 3:
**Table S3.** Candidate selection regions detected by both XP-EHH and XP-CLR tests (XLSX 73 kb) Additional file 4:
**Table S4**. High-confidence selection regions detected by both methods (XP-EHH and XP-CLR) in 2 or more populations (XLSX 41 kb) Additional file 5:
**Table S5.** Major haplotypes and their frequencies detected in the purebred lines at the high-confidence selection regions that span at least 5 SNPs in our genotype data (XLSX 12 kb) Additional file 6: Methods and results of detecting selection signatures using ZH scores. (DOCX 32 kb) Additional file 7:
**Table S7**. Candidate selection regions on autosomes detected using ZH scores (XLSX 33 kb) Additional file 8:
**Table S8.** Genes in regions detected using ZH scores that overlap with previous studies (XLSX 34 kb)

## Abbreviations

CLR: Composite likelihood ratio
EHH: Extended haplotype homozygosity
iHH: Integrated extended haplotype homozygosity
iHS: Integrated haplotype score
LD: Linkage disequilibrium
MAF: Minor allele frequency
QTL: Quantitative trait loci
XP-CLR: Cross-population composite likelihood ratio
XP-EHH: Cross-population extended haplotype homozygosity
