# Supplementary material for: Detection of genomic signatures of recent selection in commercial broiler chickens
Source: BMC Genet. 2016 Aug 26;17(1):122. doi: 10.1186/s12863-016-0430-1 (PMC5002100; doi:10.1186/s12863-016-0430-1)
Supplement: Additional file 6: — Methods and results of detecting selection signatures using ZH scores. (DOCX 32 kb) [file 12863_2016_430_MOESM6_ESM.docx]

**Supplemental File 6**

**Methods and results of detecting selection signatures using ZH scores.**

To improve our comparison with previous studies by Rubin *et al*. (2010) and Elferink *et al*. (2012) [1, 2], we estimated ZH scores (Z transformed average heterozygosity) over sliding 5-marker windows on autosomes using data from our study. For estimation of ZH scores, we used the following equations:

$$H_{i}=\frac{2\sum n_{MAJ}\sum n_{MIN}}{\left( \sum n_{MAJ}+\sum n_{MIN} \right)^{2}}$$

$${ZH}_{i}=\frac{H_{i}-\mu H}{\sigma H}$$

where $H_{i}$ is the heterozygosity of pureline $i$; $\sum n_{MAJ}$ and $\sum n_{MIN}$ are the sum of major and minor allele frequencies, respectively, within a 5-marker window; $\mu H$ is the overall average heterozygosity and $\sigma H$ is the standard deviation of all windows. Unlike the previous study by Elferink *et al*. (2012) in which allele frequencies were estimated based on the 60k SNP genotyping of DNA pools from 13 broiler lines, we estimated allele frequencies based on individual genotypes of 565 birds from 5 broiler lines. To calculate the ZH score, we first estimated allele frequencies of SNPs within each pure line, and then averaged the allele frequencies across all 5 lines.

In total, we identified 41 significant candidate selection regions with a ZH score smaller than -4, and 12 of these regions (29.3%) overlapped with regions detected by Elferink *et al*. (2012) (Table S7). Also, 81 genes could be identified in the 41 selection regions detected by ZH scores. Of these 81 genes, 22 genes overlapped with findings in Robin *et al*. (2010) and 20 genes overlapped with findings in the Elferink *et al*. study (2012). In summary, 31 genes (38%) overlapped with these previous findings (Table S8), and 11 of these 31 genes are detected in the two prior studies as well as in our study, including *IGF1, PMCH, PARPBP, NUP37, CCDC53, DRAM1, GNPTAB*, *TBXAS1, TPK1, HNF4G* and *CTK1.* Of these 11 genes, the first 8 genes are located at 55.43–56.14Mb on GGA1. This region on GGA1 is also known for QTL affecting body weight, abdominal fat and thigh muscle weight in experimental broiler chickens [3]. The most likely candidate genes under selection in this well-known QTL region could be *insulin-like growth factor 1 (IGF1)* and *pro-melanin-concentrating hormone (PMCH*)*.* Insulin-like growth factor I (IGF-I), encoded by *IGF1,* is a polypeptide hormone that stimulates the proliferation, differentiation and metabolism of myogenic cell lines in different species [4]. The importance of IGFs in growth and maintenance of various tissues has been well-established [5–10]. Previous studies showed that polymorphisms in *IGF1* were significantly associated with many important traits in broilers, such as growth, body composition and feeding traits [11–13]. On the other hand, in rat, loss of *PMCH* affected energy expenditure and resulted in a 20% lower set point for body weight [14]. Polymorphisms in *PMCH* were found to be significantly associated with growth and meat quality traits in chickens [15].

Reference:

1. Rubin C-J, Zody MC, Eriksson J, Meadows JRS, Sherwood E, Webster MT, Jiang L, Ingman M, Sharpe T, Ka S, Hallböök F, Besnier F, Carlborg O, Bed’hom B, Tixier-Boichard M, Jensen P, Siegel P, Lindblad-Toh K, Andersson L: **Whole-genome resequencing reveals loci under selection during chicken domestication.** *Nature* 2010, **464**:587–591.

2. Elferink MG, Megens H-J, Vereijken A, Hu X, Crooijmans RPM a, Groenen M a M: **Signatures of selection in the genomes of commercial and non-commercial chicken breeds.** *PLoS One* 2012, **7**:e32720.

3. Abasht B, Dekkers JCM, Lamont SJ: **Review of quantitative trait loci identified in the chicken.** *Poult Sci* 2006, **85**:2079–2096.

4. Florini JR, Ewton DZ, Coolican SA: **Growth hormone and the insulin-like growth factor system in myogenesis**. *Endocr Rev* 1996, **17**:481–517.

5. Froesch ER, Schmid C, Schwander JT, Zapf J: **Actions of insulin-like growth factors**. *Annu Rev Physiol* 1985, **47**:443–467.

6. Hammerman M: **The growth hormone-insulin-like growth factor axis in kidney**. *Am J Physiol* 1989, **257**(4 Pt 2):F503–F514.

7. Mohan S, Baylink DJ: **Impaired skeletal growth in mice with haploinsufficiency of IGF-I: genetic evidence that differences in IGF-I expression could contribute to peak bone mineral density differences.** *J Endocrinol* 2005, **185**:415–420.

8. Liu JL, Grinberg A, Westphal H, Sauer B, Accili D, Karas M, LeRoith D: **Insulin-like growth factor-I affects perinatal lethality and postnatal development in a gene dosage-dependent manner: manipulation using the Cre/loxP system in transgenic mice.** *Mol Endocrinol* 1998, **12**:1452–1462.

9. Liu JP, Baker J, Perkins AS, Robertson EJ, Efstratiadis A: **Mice carrying null mutations of the genes encoding insulin-like growth factor I (Igf-1) and type 1 IGF receptor (Igf1r).** *Cell* 1993, **75**:59–72.

10. Derek R Le, Flier JS, Underhill LH, Le Roith D: **Insulin-like growth factors**. *N Engl J Med* 1997, **336**:633–640.

11. Amills M, Jiménez N, Villalba D, Tor M, Molina E, Cubiló D, Marcos C, Francesch A, Sànchez A, Estany J: **Identification of three single nucleotide polymorphisms in the chicken insulin-like growth factor 1 and 2 genes and their associations with growth and feeding traits.** *Poult Sci* 2003, **82**:1485–1493.

12. Bennett a K, Hester PY, Spurlock DEM: **Polymorphisms in vitamin D receptor, osteopontin, insulin-like growth factor 1 and insulin, and their associations with bone, egg and growth traits in a layer--broiler cross in chickens.** *Anim Genet* 2006, **37**:283–286.

13. Zhou H, Mitchell AD, McMurtry JP, Ashwell CM, Lamont SJ: **Insulin-like growth factor-I gene polymorphism associations with growth, body composition, skeleton integrity, and metabolic traits in chickens.** *Poult Sci* 2005, **84**:212–219.

14. Mul JD, Yi C, Berg SAA Van Den, Ruiter M, Toonen PW, Elst MCJ Van Der, Voshol PJ, Ellenbroek BA, Kalsbeek A, Fleur SE, Cuppen E: **Pmch expression during early development is critical for normal energy homeostasis**. 2010.

15. Sun G, Li M, Li H, Tian Y, Chen Q, Bai Y, Kang X: **Molecular cloning and SNP association analysis of chicken PMCH gene.** *Mol Biol Rep* 2013, **40**:5049–55.
